# High Compliance to Mediterranean Diet Associates with Lower Platelet Activation and Liver Collagen Deposition in Patients with Nonalcoholic Fatty Liver Disease

**DOI:** 10.3390/nu14061209

**Published:** 2022-03-12

**Authors:** Francesco Baratta, Vittoria Cammisotto, Giulia Tozzi, Mattia Coronati, Simona Bartimoccia, Valentina Castellani, Cristina Nocella, Alessandra D’Amico, Francesco Angelico, Roberto Carnevale, Pasquale Pignatelli, Maria Del Ben

**Affiliations:** 1 Department of Clinical Internal, Anaesthesiological and Cardiovascular Sciences, Sapienza University of Rome, 00161 Rome, Italy; vittoria.cammisotto@uniroma1.it (V.C.); mattia.coronati@gmail.com (M.C.); cristina.nocella@uniroma1.it (C.N.); pasquale.pignatelli@uniroma1.it (P.P.); maria.delben@uniroma1.it (M.D.B.); 2 Division of Metabolism and Research Unit of Metabolic Biochemistry, Department of Pediatrics, IRCCS Bambino Gesù Children’s Hospital, 00146 Rome, Italy; giulia.tozzi@opbg.net; 3 Department of Medical-Surgical Sciences and Biotechnologies, Sapienza University of Rome, 04100 Latina, Italy; simona.bartimoccia@uniroma1.it (S.B.); roberto.carnevale@uniroma1.it (R.C.); 4 Department of General Surgery and Surgical Speciality “Paride Stefanini”, Sapienza University of Rome, 00161 Rome, Italy; valentina.castellani@uniroma1.it; 5 Department of Movement, Human and Health Sciences, University of Rome “Foro Italico”, 00135 Rome, Italy; a.damico@studenti.uniroma4.it; 6 Department of Public Health and Infectious Diseases, Sapienza University of Rome, 00161 Rome, Italy; francesco.angelico@uniroma1.it; 7 Mediterranea Cardiocentro, 80122 Napoli, Italy

**Keywords:** nonalcoholic fatty liver disease, Mediterranean diet, platelet activity, liver fibrosis, cardiovascular risk, thromboxane, Pro-C3

## Abstract

The Mediterranean diet (Med-Diet) is considered the most effective dietary patterns to obtain weight loss in NAFLD patients. Previous evidence suggested that Med-Diet adherence could reduce cardiovascular risk and have a beneficial effect on NAFLD severity. Aim of the study was to investigate the relationship between Med-Diet adherence, platelet activation (PA), and liver collagen deposition. The study was performed in 655 consecutive NAFLD outpatients from the PLINIO study, a prospective observational cohort study aimed to identify non-conventional predictors of liver fibrosis progression in NAFLD. PA was measured by the serum thromboxane B_2_ (TxB_2_), and liver collagen deposition by N-terminal propeptide of type III collagen (Pro-C3). Adherence to the Med-diet was investigated by a short nine-item validated dietary questionnaire. Patients with high Med-Diet adherence were older and had less metabolic syndrome and lower serum triglycerides, GGT, TxB_2_, and Pro-C3. At multivariate regression analyses, in the linear model, the Med-Diet score negatively correlated with both TxB_2_ (Beta = −0.106; *p* = 0.009) and Pro-C3 (Beta = −0.121; *p* = 0.002) and in the logistic model high adherence inversely correlated with higher TxB_2_ tertiles (II tertile: OR = 0.576, *p* = 0.044; III tertile: OR = 0.556, *p* = 0.026) and Pro-C3 tertile (III tertile: OR = 0.488, *p* = 0.013). Low consumption of red meat inversely correlated with higher TxB_2_ tertile (II tertile: OR = 0.448, *p* < 0.001, III tertile: OR = 0.567, *p* = 0.004). In conclusion, NAFLD patients with high adherence to the Med-Diet show lower PA and liver collagen deposition, suggesting a protective role of the Med-Diet against NAFLD progression and cardiovascular risk. In addition, the correlation between TxB_2_ and Pro-C3 suggests a link between NAFLD severity and cardiovascular risk.

## 1. Introduction

The Mediterranean Diet (Med-Diet) is one of the most studied diets. It is considered a lifestyle approach promoting social exchange, intercultural dialogue, and the respect of diversity [1], favoring environmentally responsible dietary approaches [2] that reduce CO_2_ consumption [3].

The Med-Diet is an eating pattern characterized by plant-based and minimally processed foods; limited consumption of sweets, red meats, and eggs; moderate wine drinking; and high-quality fats intake [4].

The Med-Diet was firstly described as the dietary pattern of Mediterranean region who suffered less frequently from cardiovascular disease in comparison to other European and American regions with different dietary habits [5]. The protective role of the Med-Diet on cardiovascular disease has been confirmed over time by different studies [6,7].

Researchers investigated the different ways by which the Med-Diet exerts its protective role. The Med-Diet is effective in weight-loss process, producing more favorable effects on glycemic control than other diets [8], reduces insulin resistance (IR) [8], is rich in antioxidant nutrients [9], reduces serum inflammation markers [10], and improves endothelial function [11]. 

Nonalcoholic fatty liver disease (NAFLD) is a growing disease affecting a quarter of the Western population [12]. NAFLD starts as lipid accumulation in at least 5% of the hepatocytes. Its natural history is characterized by the progressive development of steatohepatitis (NASH) with liver fibrosis build-up, up to the cirrhotic evolution [13]. However, liver disease progression affects only a minority of NAFLD patients, while the majority develops cardio-metabolic disease [14]. 

While a small proportion of NAFLD patients has a genetic predisposition [15], the increased NAFLD prevalence parallels the obesity epidemic [12]. Not surprisingly, the most effective therapeutic approach to reverse NAFLD is weight loss [16]. Current Guidelines recommend the Med-Diet as the most effective dietary patterns to obtain weight loss in NAFLD patients [16]. 

The Med-Diet, in NAFLD, reduces liver fat content [17] and associates with lower fibrosis severity [18]. At present, there is no evidence on the association between Med-Diet adherence and N-terminal propeptide of type III collagen (Pro-C3), a cleavage product of liver collagen III deposition during fibrillogenesis [19,20]. Together with type I, type III collagen is highly up-regulated in liver fibrosis [21]. Therefore, Pro-C3 is one of the most reliable circulating markers of liver fibrogenesis [22]. 

In addition, the Med-Diet improves IR [17,23], associates with lower oxidative stress and bacterial product intestinal translocation [24], and with a better liver enzymatic profile [25]. These effects, together with weight loss, could help reducing cardiovascular risk in patients with NAFLD. 

Platelets play a pivotal role in the atherothrombotic process leading to cardiovascular events. While the level of systemic platelet activation, and its impact on cardiovascular risk, has been poorly investigated in NAFLD patients, platelet activity, in the liver context, has been correlated to NAFLD severity in both human and experimental models [26,27]. In humans, the number of platelets identified within liver sinusoids correlates with the NAFLD activity score (NAS) and the presence of ballooning degeneration [26]. In a murine model, platelet adhesion and activation, but not platelet aggregation, were identified as crucial for NASH onset and progression [27]. Regarding the impact of the Med-Diet on platelet activity, several studies proved a negative association between platelet activity and adherence to the Med-Diet [28,29]. However, there is no evidence on the effects of the Med-Diet on platelet function in NAFLD patients. 

The evidence above discussed, suggest a possible influence of the Med-Diet on both levels of liver fibrogenesis and platelet activation in NAFLD patients. Therefore, the aim of the present study is to investigate the correlation between Med-Diet adherence, circulating platelet activation markers, and liver collagen deposition.

## 2. Materials and Methods

This is a post hoc analysis of the PLINIO study (Progression of Liver Damage and Cardiometabolic Disorders in Non-alcoholic Fatty Liver disease: an Observational Cohort study. ClinicalTrials.gov Identifier: NCT04036357) including 655 consecutive NAFLD patients. The Plinio study is a prospective observational cohort study aimed to detect non-conventional factors associated with the progression of liver fibrosis in patients with NAFLD.

We enrolled patients referred to the Day Service of Internal Medicine and Metabolic Disorders of the Policlinico Umberto I University Hospital in Rome with at least one out of the following cardio-metabolic diseases: metabolic syndrome (MetS), overweight/obesity, type 2 diabetes, arterial hypertension, dyslipidemia, or atrial fibrillation (AF). Exclusion criteria were: alcohol abuse (>20 g/day in women and >30 g/day in men) confirmed by the use of Alcohol Use Disorders Identification Test, AUDIT [30]; present or past history of hepatitis B or hepatitis C virus; history of autoimmune hepatitis; presence of other chronic liver diseases; diagnosis of malignancy; use of steatogenic drug (e.g., amiodarone).

Patients presenting inclusion criteria and none of the exclusion criteria underwent liver ultrasound (US). Only patients with evidence of liver steatosis were included in the present analysis. 

Anthropometric data (i.e., waist circumference and body mass index, BMI), and clinical and drug history were registered. The presence of cardiovascular and metabolic risk factors was defined according to international guidelines [31,32,33]. Routine clinical and biochemical evaluations were performed.

Liver US scanning was performed to assess the presence of steatosis. All US were performed by the same operator who was blinded to laboratory values using a GE Vivid S6 apparatus equipped with a convex 3.5 MHz probe; fatty liver was defined according to Hamaguchi score. Hamaguchi score was calculated as follow: echoes intensity arising from liver parenchyma with presence or absence of liver-kidney contrast (0–3 points), clarity of diaphragm visualization (0–2 points), and clarity of liver blood vessel structure (0–1 point). A score of 0 points excluded the presence of steatosis, a score ≥ 1, in presence of increased liver parenchyma echoes, was considered diagnostic for the presence of liver steatosis [34]. Aspartate aminotransferase-to-platelet ratio index (APRI), a non-invasive marker of advanced fibrosis, was calculated as follows

ASTAST(Uppe Normal Limit)Platelets count (109l)∗100

Values of APRI > 0.7 were considered positive for predicting significant hepatic fibrosis [35,36].

The study was conducted according to the 2008 update of the Declaration of Helsinki. All patients subscribed informed written consent. Study protocol was approved by the local ethical board of Sapienza-University of Rome (Ref. 2277 prot. 873/11). All co-authors had access to the study data and had reviewed and approved the final manuscript.

### 2.1. Med-Diet Questionnaire

Adherence to the Med-Diet was investigated by a short 9-item validated and previously described dietary questionnaire. It assigns 1 point for: (1) olive oil (≥1 spoon/day, i.e., tablespoon of 10 gr of olive oil); (2) fruit (≥1 serving/day); (3) vegetables or salad (≥1 serving/day); (4) both fruit (≥1 serving/day) and vegetables (≥1 serving/day); (5) legumes (≥2 servings/week); (6) fish (≥3 servings/week); (7) wine (≥1 glass/day, ≤20 g for women and ≤30 g for men; (8) meat (<1 serving/day); (9) (both white bread (<1/day) and rice (<1/week)) or whole-grain bread (>5/week). A cumulative score ranging from 0 to 9 points was obtained. Patients were defined as low (0–2 pts), intermediate (3–5 pts), or high adherers to the Med-Diet (7–9 points) [37].

### 2.2. Serum TxB_2_ Assay

Serum Thromboxane (Tx) B_2_ was analyzed by an ELISA commercial kit (Cusabio, Houston, TX, USA), according to manufacturer instructions. The values were expressed as pg/mL. Intra- and inter-assay coefficients of variation for TxB_2_ were <8% and <10%, respectively.

### 2.3. Pro-C3 Assay 

The detection of N-terminal propeptide of type III collagen (Pro-C3) was performed in serum samples by using Human PIIINP enzyme-linked immunosorbent assay (ELISA) (Novus Biological, Oceanside, CA, USA). Both intra- and inter-assay coefficients of variation for PIIINP were less than 10%.

### 2.4. Statistical Analysis 

Categorical variables were reported as percentages; normal and non-normal continuous variables were reported as means ± SD and median and interquartile range [25–75th], respectively. Comparisons between groups were analyzed by ANOVA test and Bonferroni post hoc analysis or Kruskal–Wallis and Mann–Whitney tests (for continuous variables) or chi-square test (for categorical variables). Univariate analyses were conducted using Pearson correlation (r) analysis and Spearman correlation (rS) analysis for normal and non-normal variables, respectively. 

Multivariate linear regression analyses were performed to test factors associated with TxB_2_ and Pro-C3. Non normal variables were log-transformed. 

Multivariate logistic regression analyses were performed to test factors associated to TxB_2_ and Pro-C3. Lower tertiles were considered as reference ones. 

A value of *p* < 0.05 was considered as statistically significant. All analyses were performed with SPSS V.27 and JMP software version 15-SAS Institute. 

## 3. Results

### 3.1. Patients’ Charachteristics According to Med-Diet Adherence

We included 655 NAFLD patients from the PLINIO study. Mean age was 54.9 ± 11.7 years and 38.6% were female. The median Med-Diet Score was 5 (4–6) points. According to score cut offs 10.2%, 74.3%, and 15.4% of the participants had low, intermediate, and high adherence to the Med-Diet, respectively. Patients with low adherence, as compared to those with high adherence, were younger, had higher prevalence of metabolic syndrome (70.1% vs. 52.5%, *p* = 0.022), and higher median value of triglycerides (164.0 (114.0–212.0) vs. 127.0 (103.0–163.0) mg/dl, *p* = 0.007) and of GGT (30.5 (19.7–44.2) vs. 23.0 (17.0–33.2) UI/l, *p* = 0.028) (Table 1). No differences were found in antiplatelet therapy according to the Med-Diet adherence group (Table 1 and Table S1).

### 3.2. Med-Diet Score and TxB_2_

Mean TxB_2_ was 184.8 ± 32.0 pg/mL and decreased progressively from subjects with low Med-Diet adherence to those with intermediate and high adherence (191.4 ± 32.6 vs. 185.4 ± 33.2 vs. 177.5 ± 24.1 pg/mL, respectively, *p* = 0.015). No differences in mean TxB_2_ were found according to specific antiplatelet drugs (Table S2). At univariate analysis, the Med-Diet score correlated with TxB_2_ (rS = −0.100; *p* = 0.010). After correction for sex, total-c/HDL-c, glycaemia, BMI, and APRI, an independent negative association was demonstrated between serum TxB_2_ and the Med-Diet score (Beta = −0.10; *p* = 0.009), in addition to age (Beta = −0.09; *p* = 0.002) and APRI (Beta = 0.25; *p* < 0.001) (Table 2).

At multivariate logistic regression analysis, high adherence to the Med-Diet (score > 6 pts) associated with TxB_2_ (III tertile vs. I tertile: OR = 0.55, *p* = 0.023 and II tertile vs. I tertile: OR = 0.56, *p* = 0.038) after correction for age > 65 years, sex, metabolic syndrome, and antiplatelet therapy. in addition, APRI > 0.7 associated with TxB_2_ (III tertile vs. I tertile: OR = 2.39, *p* = 0.039) (Table 3). 

**Figure 1:**
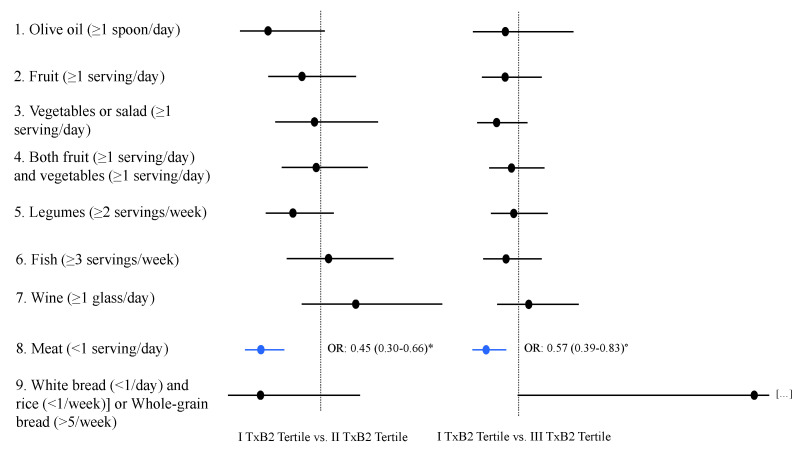
Multivariate regression analyses of factors associating with each Mediterranean diet food score items. (TxB_2_ odds ratio and relative confidence interval were adjusted for age > 65 years, sex, metabolic syndrome, antiplatelet therapy, APRI > 0,7). * *p* < 0.001; ° *p* = 0.004.

Performing additional multivariate analyses using each food score items instead of the Med-Diet adherence score, TxB_2_ (II vs. I tertile: OR = 0.45, *p* < 0.001, III vs. I tertile: OR = 0.57, *p* = 0.004) correlated with low meat consumption (meat < 1 serving/day) after correction for age > 65 years, sex, metabolic syndrome, antiplatelet therapy and APRI > 0.7 (Figure 1).

### 3.3. Med-Diet Score and Pro-C3

In the whole population the mean Pro-C3 was 7.2 ± 2.6 ng/mL and decreased progressively from subjects with low Med-Diet adherence to those with intermediate and high adherence (7.8 ± 3.0 vs. 7.3 ± 2.7 vs. 6.3 ± 1.7 ng/mL, respectively, *p* < 0.001). No differences in mean Pro-C3 were found according to specific antiplatelet drugs (Table S2).

At univariate analysis, a negative correlation was observed between the Med-Diet score and Pro-C3 (rS = −0.135; *p* < 0.001). After correction for age, sex, total-c/HDL-c, glycaemia, and BMI, Pro-C3 associated with the Med-Diet score (Beta = −0.12; *p* = 0.002) and APRI (Beta = 0.10, *p* = 0.008) (Table 4).

At multivariate logistic regression analysis, high adherence to the Med-Diet (score > 6 pts) was associated with Pro-C3 (III vs. I tertile: OR = 0.48, *p* = 0.010) after correction for sex, metabolic syndrome, and antiplatelet therapy. In addition, age ≥ 65 years associated with Pro-C3 tertiles (III vs. I tertile: OR = 0.60, *p* = 0.038; II vs. I tertile: OR = 0.57, *p* = 0.023) (Table 5). 

When in the multivariate analyses, the individual food items scores were included as independent variables instead of the Med-Diet score, no statistically significant associations with Pro-C3 tertiles were observed (data not showed).

At univariate analysis, TxB_2_ and Pro-C3 were positively correlated (r = 0.110; *p* = 0.005). The correlation was also confirmed after adjustment for age and sex (Beta = 0.106; *p* = 0.007).

## 4. Discussion

The beneficial effects of the Med-Diet were widely proved. Previous studies have demonstrated anti-inflammatory [38] and antioxidant effects in both the general population [39] and NAFLD patients [24]. Instead, this is the first study proving an inverse correlation between Med-Diet adherence, circulating platelet activation marker, and liver collagen deposition in patients with NAFLD. 

Platelet activation is a key moment in atherothrombotic processes leading to CVD. CVD is the first cause of morbidity and mortality in patients with NAFLD [40,41]. While impaired lipid profile [42], diabetes [43], endothelial dysfunction [44], and oxidative stress [45,46] were investigated as possible risk factors in NAFLD patients, few studies have addresses platelet activation as a cardiovascular risk marker. Some authors have suggested mean platelet volume (MPV), a reliable marker of platelet hyperactivity and CV risk [47], as a risk marker in NAFLD patients, and have included MPV in a risk score for CV events in these patients [43]. 

To the best of our knowledge, this is the first study reporting TxB_2_ levels in a large population of NAFLD patients and investigating the relationship between TxB_2_ and adherence to the Med-Diet in this setting. Serum TxB_2_, produced by the non-enzymatic hydration of thromboxane A2, which is produced in turn by platelet cyclooxygenase 1 [48], reflects the body production of TxB_2_ and is a reliable marker of platelet activation ex vivo [49]. Whereas in other settings, such as atrial fibrillation [50] and pneumonia [51], the relationship between platelet activation, the Med-Diet, and cardiovascular events is well described, to date, there are no prospective data on the association between Med-Diet adherence, TxB_2_, and cardiovascular events in NAFLD patients.

Our data also demonstrate for the first time that the lower the adherence to the Med-Diet, the higher the values of serum Pro-C3, confirming that the Med-Diet might be associated with a less severe NAFLD. Serum Pro-C3, cleaved during collagen III deposition, is a circulating marker of fibrogenesis that progressively increase with fibrosis stage and NAFLD activity score (NAS) [19]. Of interest, we found a direct correlation between Pro-C3 and APRI, a noninvasive score of liver fibrosis. In accordance with our data, a previous study form Kontogianni MD et al. showed an inverse correlation between Med-Diet adherence and both liver fibrosis (diagnosed with histology or liver stiffness measurement) and NASH diagnosis [52]. 

When we analyzed the single food items of the Med-Diet score, we found that none of them associated with higher values of Pro-C3, suggesting a role of the Med-Diet as a whole in influencing collagen liver deposition. 

Instead, performing a further analysis including TxB_2_, we found that lower consumption of meat (less than 1 serving/week) associates with lower levels of serum TxB_2,_ suggesting a less severe NAFLD in patients who eat less meat. Differently, Pignatelli et al. [29] demonstrated that, in atrial fibrillation patients, high olive oil consumption inversely correlated with the level of TxA_2_ production. 

Our data could partially be explained by previous findings. Ahmad M.I. and colleagues [53], linked diets rich in red meat and NAFLD risk via the high oxidative stress induced by this dietary pattern. Interestingly, we previously demonstrated, although in different settings, the association between low adherence to the Mediterranean diet and both high circulating level of lipopolysaccharide (LPS) and NADPH oxidase 2 (NOX2), markers of gut bacterial products translocation and systemic oxidative stress, respectively [24,54]. Oxidative stress is a well-known factor inducing platelet activation [55] and could represent the link between meat consumption and impaired platelet activation.

In addition, we found an independent association between TxB_2_ and Pro-C3 that might confirm a role of platelet activity in liver damage as also supported by the association between TxB_2_ and APRI. 

Finally, this study confirms, as previously observed [23], that older patients adhere more to the Med-Diet than younger ones. This finding is in agreement with that observed in other Italian study populations [56]. The trend of Mediterranean youth to prefer Western dietary habits was widely discussed in the literature in the last years [57,58] and was summarized in a systematic review [59]. This has been related to the more frequent habits to have launch away from home because of work [57]. Interestingly, in the last 2 years, during COVID19 pandemic, young adults [18–30 years] paid more attention to eating Med-Diet foods [60]. 

Our findings have clinical implication, adding evidence on the beneficial role of the Med-Diet in preventing both NAFLD progression and cardiovascular disease in this setting. Previous studies already proved that high adherence to the Med-Diet induces effective weight loss, liver fat, and total mass fat reduction, liver stiffness, and ALT improvement [61,62,63]. Of note, the Med-Diet score we used was validated in a study aimed to the quantitative estimation of adherence to a cardioprotective Mediterranean diet [37], giving even more strength to the association between Med-Diet adherence and TxB_2_ we found. 

Our study has also some limitations. Its observational and cross-sectional design does not allow the establishment of a cause–effect relationship between Med-Diet adherence, impaired platelet activity, and liver collagen deposition. In addition, the Med-Diet short questionnaire we used was not designed to investigate single nutrient intake and we therefore could not investigate the association between serum TxB_2_ and Pro-C3 with specific nutrients. Finally, despite US scanning being the most suitable imaging test to diagnose liver steatosis, due its cost-effectiveness and high specificity, it has a low diagnostic sensitivity for mild steatosis.

## 5. Conclusions

NAFLD patients high adhering to the Med-Diet show lower platelet activation and liver collagen III deposition, suggesting a protective role of the Med-Diet against NAFLD progression and cardiovascular risk. In addition, the positive correlation between serum TxB_2_ and Pro-C3 may suggest a link between NAFLD severity and the level of cardiovascular risk.

## Figures and Tables

**Table 1:** Patients’ characteristics according to the adherence to Mediterranean Diet.

	Adherence to Mediterranean Diet				
	Low (Score 0–2 pts) (*n* = 67)	Intermediate (Score 3–6 pts) (*n* = 487)	High (Score 7–9 pts) (*n* = 101)	p_among all_	p_low vs. high_
Age (years)	51.3 ± 13.1	55.3 ± 11.4	55.2 ± 11.6	0.028	0.092
Female (%)	34.3	40.7	31.7	0.181	0.721
BMI (Kg/m^2^)	31.1 ± 5.4	30.5 ± 5.0	29.7 ± 4.3	0.144	0.185
Obesity (BMI ≥ 30 Kg/m^2^) (%)	53.7	49.3	41.6	0.248	0.122
Metabolic syndrome (%)	70.1	61.1	52.5	0.063	0.022
Waist circumference (cm)	108.2 ± 12.8	107.2 ± 12.0	105.8 ± 9.9	0.392	0.581
Diabetes (%)	25.4	29.2	27.7	0.797	0.736
Glycaemia (mg/dL)	107.0 ± 38.4	106.1 ± 27.7	102.9 ± 19.4	0.531	1.000
Antiplatelet drugs (%)	20.9	15.6	11.9	0.288	0.114
Statin use (%)	40.3	39.0	33.7	0.566	0.381
Arterial hypertension (%)	56.7	61.4	55.4	0.456	0.871
Total cholesterol (mg/dL)	202.1 ± 43.2	196.5 ± 42.1	195.5 ± 38.7	0.554	0.950
HDL (mg/dL)	46.4 ± 10.7	47.9 ± 14.1	47.5 ± 12.7	0.702	1.000
Total cholesterol/HDL	4.6 ± 1.4	4.4 ± 1.7	4.4 ± 1.3	0.690	1.000
Triglycerides (mg/dL)	164.0 (114.0–212.0)	137.0 (103.0–183.0)	127.0 (103.0–163.0)	0.026	0.007
GGT (UI/L)	30.5 (19.7–44.2)	28.0 (17.0–42.0)	23.0 (17.0–33.2)	0.074	0.028
AST (UI/L)	21.5 (18.0–28.2)	22.0 (18.0–29.0)	20.0 (17.0–26.7)	0.225	0.265
ALT (UI/L)	32.0 (20.0–43.0)	28.0 (20.0–44.0)	25.0 (18.0–35.0)	0.093	0.054
Platelets	250.9 ± 60.4	237.6 ± 63.0	230.4 ± 52.3	0.109	0.108
AST-to-Platelet ratio	0.3 (0.2–0.3)	0.3 (0.2–0.4)	0.3 (0.2–0.3)	0.465	0.900
TxB_2_ (pg/mL)	191.4 ± 32.6	185.4 ± 33.2	177.5 ± 24.1	0.015	0.017
Pro-C3 (ng/mL)	7.8 ± 3.0	7.3 ± 2.7	6.3 ± 1.7	<0.001	<0.001

BMI: body mass index; HDL: high-density lipoprotein; GGT: gamma-glutamyltransferase; AST: aspartate aminotransferase; ALT: alanine aminotransferase; TxB_2_: thromboxane B_2_; Pro-C3: N-terminal propeptide of type III collagen. Normal variables were expressed as mean ± SD; differences were tested using ANOVA test and Bonferroni post-hoc analysis; non-normal variables were expressed as median (25th–75th), differences were tested using Kruskal–Wallis and Mann–Whitney.

**Table 2:** Multivariate linear regression analysis of factors associated with TxB_2_.

Panel A	B	S.E.	Beta	*p*
Age	−0.26	0.11	−0.09	0.022
Female sex	2.73	2.64	0.04	0.300
Total-c/HDL-c	0.58	1.19	0.02	0.625
Glycaemia	0.02	0.05	0.02	0.661
BMI	−0.06	0.25	−0.01	0.809
Med-Diet score	−1.93	0.74	−0.10	0.009
Triglycerides *	−8.19	7.53	−0.05	0.277
APRI *	35.38	5.47	0.25	<0.001

Total-c: total cholesterol; HDL-c: high-density lipoprotein cholesterol; BMI: body mass index; TxB_2_: thromboxane B_2_; APRI: AST-to-platelets ratio; Pro-C3: N-terminal propeptide of type III collagen. * Non-normal variables were log-transformed.

**Table 3:** Multivariate logistic regression analysis of factors associated TxB_2_ tertiles.

Panel A	III TxB_2_ Tertile *		II TxB_2_ Tertile *	
	Odds Ratio (95% C.I for OR)	*p*	Odds Ratio (95% C.I for OR)	*p*
Age ≥ 65 years	0.99 (0.62–1.57)	0.956	0.50 (0.30–0.85)	0.010
Female Sex	1.20 (0.81–1.78)	0.364	1.42 (0.95–2.13)	0.087
Metabolic syndrome	0.92 (0.62–1.36)	0.682	1.34 (0.89–2.01)	0.166
Antiplatelet therapy	0.91 (0.52–1.59)	0.734	1.44 (0.84–2.47)	0.182
APRI > 0.7	2.39 (1.05–5.47)	0.039	1.13 (0.42–3.06)	0.813
High adherence to Med-Diet	0.55 (0.32–0.92)	0.023	0.56 (0.33–0.97)	0.038

C.I: confidence interval; OR: odds ratio; Total-c: total cholesterol; HDL-c: high-density lipoprotein cholesterol; BMI: body mass index; TxB_2_: thromboxane B_2_; APRI: AST-to-platelets ratio; Pro-C3: N-terminal propeptide of type III collagen. * I TxB_2_ tertile was considered as reference.

**Table 4:** Multivariate linear regression analyses of factors associated with Pro-C3.

	B	S.E.	Beta	*p*
Age	−0.01	0.01	−0.05	0.273
Female sex	0.12	0.22	0.02	0.593
Total-c/HDL-c	−0.15	0.10	−0.08	0.133
Glycaemia	0.00	0.00	0.03	0.469
BMI	0.03	0.02	0.05	0.214
Med-Diet score	−0.19	0.06	−0.12	0.002
Triglycerides *	−0.14	0.64	−0.01	0.821
APRI *	1.22	0.46	0.10	0.008

Total-c: total cholesterol; HDL-c: high-density lipoprotein cholesterol; BMI: body mass index; TxB_2_: thromboxane B_2_; APRI: AST-to-platelets ratio; Pro-C3: N-terminal propeptide of type III collagen. * Non-normal variables were log-transformed.

**Table 5:** Multivariate logistic regression analyses of factors associated with Pro-C3 tertiles.

	III Pro-C3 Tertile *		II Pro-C3 Tertile *	
	Odds Ratio (95% C.I for OR)	*p*	Odds Ratio (95% C.I for OR)	*p*
Age ≥ 65 years	0.60 (0.37–0.97)	0.038	0.57 (0.35–0.92)	0.023
Female Sex	1.23 (0.82–1.84)	0.307	1.44 (0.97–2.15)	0.073
Metabolic Syndrome	1.00 (0.67–1.50)	0.993	0.89 (0.59–1.34)	0.579
Antiplatelet therapy	0.99 (0.58–1.69)	0.982	0.73 (0.42–1.27)	0.271
APRI > 0.7	1.66 (0.75–3.65)	0.208	0.62 (0.23–1.65)	0.336
High Adherence to Med-Diet	0.48 (0.27–0.84)	0.010	0.89 (0.54–1.48)	0.563

C.I: confidence interval; OR: odds ratio; Total-c: total cholesterol; HDL-c: high-density lipoprotein cholesterol; BMI: body mass index; TxB_2_: thromboxane B_2_; APRI: AST-to-platelets ratio; Pro-C3: N-terminal propeptide of type III collagen. * I Pro-C3 tertile was considered as reference.

## Data Availability

The data presented in this study are available on request from the corresponding author.

## Supplementary Materials

The following supporting information can be downloaded at: https://www.mdpi.com/article/10.3390/nu14061209/s1; Table S1: Multivariate linear regression analysis of factor associated with Mediterranean Diet Score, including both TxB_2_ and Pro-C3; Table S2: Multivariate logistic regression analysis of factors associated with high adherence to Mediterranean Diet (score > 6 pts), including both TxB_2_ and Pro-C3 tertiles.
