# Supplementary material for: High Compliance to Mediterranean Diet Associates with Lower Platelet Activation and Liver Collagen Deposition in Patients with Nonalcoholic Fatty Liver Disease
Source: Nutrients. 2022 Mar 12;14(6):1209. doi: 10.3390/nu14061209 (PMC8952322; doi:10.3390/nu14061209)
Supplement: Supplementary file 1 [file nutrients-14-01209-s001.zip › nutrients-1609007-supplementary.pdf]

Table S1. Antiplatelet drug use according to Med-Diet-Adherence.

	Adherence to Mediterranean Diet				
	Low (Score 0-2 pts) (n=67)	Intermediate (Score 3-6 pts) (n=487)	High (Score 7-9 pts) (n=101)	$p_{among\ all}$	$p_{low\ vs\ high}$
**Aspirin (%)**	19.4	14.0	11.9	0.376	0.191
**Clopidogrel (%)**	1.5	1.2	0	0.515	0.399
**Ticlopidine (%)**	1.5	1.0	0	0.537	0.399

Table S2. Mean TxB_2 and Pro-C3 according to aspirin, clopidogrel and ticlopidine use.

	**Aspirin**		**p**	**Clopidogrel**		**p**	**Ticlopidine**		**p**
	Yes (n=93)	No (n=562)		Yes (n=7)	No (n=648)		Yes (n=6)	No (n=649)	
**TxB_2 (pg/ml)**	184.0 ± 32.7	184.9 ± 32.0	0.809	188.9 ± 15.4	184.8 ± 32.2	0.735	182.9 ± 4.4	184.8 ± 32.2	0.882
**Pro-C3 (ng/ml)**	7.2 ± 2.9	7.2 ± 2.6	0.836	7.6 ± 2.8	7.2 ± 2.6	0.686	8.7 ± 3.5	7.2 ± 2.6	0.149
